# The X-Rays in Phthisis Pulmonalis

**Published:** 1908-10-03

**Authors:** 


### Medicine.

### THE X-RAYS IN PHTHISIS PULMONALIS.

The only clinching proof of the existence of phthisis pulmonalis is the detection of tubercle bacilli in the sputum: but there are cases of suspected phthisis in which no sputum is obtainable. A very careful physical examination of the apices may reveal the few suspicious râles at the end of a deep inspiration or after a cough, these râles persisting from day to day, although inspection, palpation, and percussion show no other abnormality. A persistent râle at an apex is always a cause for anxiety, and probably it is the very earliest of all the physical signs to which phthisis may give rise. Nevertheless there are not a few cases in which tubercle bacilli are present in the sputum, and in which there may have been hæmoptysis, and yet there is not even a suspicious râle to be detected. In other words, the absence of all abnormal physical signs is no guarantee of the absence of phthisis, and this no matter how careful and experienced the examiner may be. This being so, any additional means of detecting phthisis early is to be welcomed, and such a means is to be found in the *x*-rays.

This is not to say that the *x*-rays should be employed in examining the lung in every case of phthisis, but the accumulation of a great quantity of evidence proves beyond doubt that, properly interpreted, the *x*-rays are as valuable a means of physical examination of the lungs and pleura as any we possess. It is quite clear, of course, that all the shadows and speckles seen in the affected lungs under the *x*-rays are not necessarily due to phthisis, for a resolving apical lobar pneumonia, for example, may be indistinguishable in a skiagram from tuberculous bronchopneumonia or fibrosis. The interpretation of all shadows seen has to be made from other points in the case—history, symptoms, and so forth—as with other physical signs. If, however, a patient is suspected to have early phthisis, and if neither sputum nor ordinary physical examination of the chest serve to settle any doubt there may be, then it is possible that the *x*-rays will do so. It is well established that in some cases *x*-ray evidence strongly pointing to phthisis can be obtained before any abnormal physical signs exist. Further than this, in the large majority of cases in which definite signs exist at one apex only, *x*-rays will show shadows indicating that the other apex is involved.

There is some difference of opinion as to whether absence of *x*-ray signs at the apices can be taken to mean absence of phthisis. Enthusiasts believe so firmly in the power of the *x*-rays to detect even the slightest lung mischief that they would say: no *x*-ray signs, no phthisis. This, however, is probably going too far. It is even probable that in some instances there may be positive evidence of phthisis on ordinary physical examination in a case in which the *x*-rays show no abnormality. In such a case the positive evidence of the ordinary physical signs is of much greater weight than is the negative evidence of the *x*-rays; but if in a suspected case the *x*-rays show signs at one or other apex, the probability of existent phthisis is considerable, even though there are no other abnormal physical signs whatever.

There are, some say, five *x*-ray signs of phthisis, but really these may be reduced to the following three, namely: (1) Some alteration in the movements of the diaphragm; (2) some deficiency in the clearness of one or other apex on deep breathing; (3) some mottling or speckling in the picture of the affected lung-apex. The other two points are: (*a*) alterations in the shape of the chest, especially such as cause asymmetry. These are too common to be of much value; they occur in many other conditions besides phthisis, and they do not occur in ordinary cases of phthisis until the latter is considerably advanced or fibrotic. (*b*) The cardiac shadow is often more vertical than it should be; this, it is supposed, is due to the puckering and fibrosis of one or other pleura drawing the base of the heart upwards, and so tilting the whole organ into a more vertical position. This might occur in a case of local pleural fibrosis of very old date, the result of some obsolete pleurisy.

Both halves of the diaphragm are to be very clearly seen under the *x*-rays, and their movements can be very accurately followed. If, in a suspected case of phthisis, it is found that one side is restricted in its excursions, whereas the other moves perfectly well, the suspicion of disease is converted into a certainty. The reverse is not true; that is to say, free movement of both sides of the diaphragm does not exclude phthisis; but it is not at all uncommon to derive a great deal of assistance in diagnosis from these unequal movements.

Deficiency of apical clearness on deep inspiration is a sign of great value. Dr. C. Thurstan Holland says: "I have seen cases in which both sides of the diaphragm moved fairly well in ordinary and in deep respiration where no distinct abnormal shadowing was made out, and where everything appeared normal, but when a deep breath was taken one apex would light up brilliantly and the other by comparison remain opaque. In other cases, whilst the rest of the lung areas lit up in deep inspiration, the translucency of neither apex altered at all. Again and again I have seen this appearance on the screen, and if it exists in a suspected case I think it is quite enough to go upon."

Mottling or speckling of the affected apex is not as a rule to be detected until the phthisis is pretty decided. Even with cavities in the lung, however, one knows perfectly well that the ordinary methods of physical examination may fail to detect any disease, and therefore a mottled apical shadow—due to scattered areas of fibrosis or consolidation, or cavitation with pus in the cavities—may be a sign of hitherto undiagnosed though suspected phthisis. The mottling shows best in skiagrams, whereas the deficient movement of the diaphragm and the deficient clearing of the apices on deep breathing show only on the screen. It is necessary, therefore, actually to see the patient under the *x*-rays for an opinion to be formed.

